# Signal Transduction through CsrRS Confers an Invasive Phenotype in Group A *Streptococcus*

**DOI:** 10.1371/journal.ppat.1002361

**Published:** 2011-10-27

**Authors:** Hien J. Tran-Winkler, John F. Love, Ioannis Gryllos, Michael R. Wessels

**Affiliations:** 1 Division of Infectious Diseases, Children's Hospital Boston, Boston, Massachusetts, United States of America; 2 Harvard Medical School, Boston, Massachusetts, United States of America; 3 Division of Infectious Diseases, Beth Israel Deaconess Medical Center, Boston, Massachusetts, United States of America; University of Birmingham, United Kingdom

## Abstract

The CsrRS (or CovRS) two component system controls expression of up to 15% of the genome of group A *Streptococcus* (GAS). While some studies have suggested that the sensor histidine kinase CsrS responds to membrane perturbations as a result of various environmental stresses, other data have implicated the human antimicrobial peptide LL-37 and extracellular Mg^2+^ as specific signals. We now report that Mg^2+^ and LL-37 have opposite effects on expression of multiple genes that are activated or repressed by the transcriptional regulator CsrR. Using a GAS isolate representative of the recently emerged and widely disseminated M1T1 clone implicated in severe invasive disease, we found marked up-regulation by CsrRS of multiple virulence factors including pyrogenic exotoxin A, DNase Sda1, streptolysin O, and the hyaluronic acid capsular polysaccharide, among others. Topology and surface protein labeling studies indicated that CsrS is associated with the bacterial cell membrane and has a surface-exposed extracellular domain accessible to environmental ligands. Replacement of a cluster of three acidic amino acids with uncharged residues in the extracellular domain of CsrS abrogated LL-37 signaling and conferred a hyporesponsive phenotype consistent with tonic activation of CsrS autokinase activity, an effect that could be overridden by mutation of the CsrS active site histidine. Both loss- and gain-of-function mutations of a conserved site in the receiver domain of CsrR established an essential role for lysine 102 in CsrS-to-CsrR signal transduction. These results provide strong evidence that Mg^2+^ and LL-37 are specific signals that function by altering CsrS autokinase activity and downstream phosphotransfer to CsrR to modulate its activity as a transcriptional regulator. The representation of multiple antiphagocytic and cytotoxic factors in the CsrRS regulon together with results of in vitro phagocytic killing assays support the hypothesis that CsrRS mediates conversion of GAS from a colonizing to an invasive phenotype in response to signaling by host LL-37.

## Author Summary

Group A Streptococcus (*S. pyogenes* or GAS) is exclusively a human pathogen that can inhabit the human throat as a harmless commensal, cause localized, self-limited infection in the form of pharyngitis or strep throat, or invade local tissues or the bloodstream to produce life-threatening disease states such as necrotizing fasciitis or streptococcal toxic shock. We present evidence that the GAS CsrRS (or CovRS) two component system governs the transition from a colonizing to an invasive phenotype by transducing a specific signal from the antimicrobial peptide LL-37 that is secreted as part of the human innate immune response to GAS infection. We show that LL-37 signaling requires specific domains of both the CsrS sensor kinase and the CsrR response regulator, and that signaling results in a coordinated and marked increase in expression of multiple bacterial factors that confer resistance to phagocytic killing, a hallmark of GAS virulence.

## Introduction

Human beings are thought to be the principal if not exclusive host for group A *Streptococcus* (*S. pyogenes*, GAS). The organism's primary environmental niche is the human pharynx where GAS can colonize the epithelium without evoking any clinical symptoms, or it can produce local inflammation and symptomatic streptococcal pharyngitis [1], [2]. GAS also causes impetigo, a superficial skin infection, and, less commonly, severe invasive infections such as necrotizing fasciitis, bacteremia, and streptococcal toxic shock [3], [4]. The regulated expression of a variety of gene products enhances GAS survival in the human host through a dynamic process of adaptation to stresses that may change depending on the precise anatomic location of the bacteria in the body, environmental factors, and engagement of host defense mechanisms [5], [6].

Two component regulatory systems (TCS) play an important role in such dynamic adaptation of many bacteria to changing environmental conditions [7], [8]. CsrRS (also called CovRS) is the most extensively characterized TCS in GAS. First identified as a regulator of the *has* operon that encodes the enzymes required for synthesis of the hyaluronic acid capsular polysaccharide, CsrRS has since been shown to affect expression of as much as 15% of the GAS genome including genes encoding many virulence factors [9]–[12]. Genetic evidence and similarity to TCS in other species have suggested that CsrS is a sensor histidine kinase whose phosphorylation state is influenced by environmental signals, while CsrR is a transcriptional regulator whose activity at target promoters is controlled by phosphorylation. It is presumed, but not proven, that phosphorylation of CsrR results from phosphotransfer from CsrS. It has also been proposed that CsrS has a phosphatase activity and can dephosphorylate CsrR [13]. Transcriptional profiling of CsrR- or CsrRS-mutants has indicated that CsrR acts primarily, although not exclusively, as a repressor of gene expression, as mutants exhibit increased expression of most CsrRS-regulated genes, and phosphorylation of CsrR in vitro enhances its binding to regulated promoters [9], [10], [14], [15].

While it is clear that CsrRS influences expression of many important GAS products, a unifying explanation of the adaptive role of the CsrRS system is still unproven. One proposal is that CsrRS represents a system to detect and respond to a variety of environmental stresses, such as elevated temperature, acidic pH, and high osmolarity, all of which might result in alterations in physical properties of the bacterial cell membrane and consequent signaling through CsrS [13]. An alternative model is that CsrS recognizes specific ligands, and that interaction of these ligands with its extracellular domain (ECD) results in changes in CsrS autokinase activity and/or phosphatase activity for CsrR. The latter model is based on the findings that increased concentrations of extracellular Mg^2+^ result in widespread down-regulation of CsrR-repressed genes, an effect dependent on a functional CsrS and not reproduced by other cations [11], [16]. Thus, Mg^2+^ may serve as a specific stimulus for activation of CsrS kinase activity with downstream phosphorylation of CsrR. The human antimicrobial peptide LL-37 has been shown to have effects on CsrRS signaling opposite to those of elevated Mg^2+^. Concentrations of LL-37 far below those that inhibit GAS growth were shown to stimulate increased expression of the *has* operon and three other CsrR-repressed genes in a CsrS-dependent fashion [17]. While these two models are not necessarily mutually exclusive, it is difficult to reconcile LL-37 signaling with a model of non-specific membrane perturbation since the effects of LL-37 on gene expression were not reproduced by a broad range of doses of other antimicrobial peptides, including other cathelicidins, of similar or greater antibacterial potency [17].

The highly specific effect of LL-37 to stimulate up-regulation of CsrR-repressed genes suggests that CsrRS functions to detect and counteract host immune effectors that mediate bacterial clearance from the infected host. Circumstantial evidence for such a role comes from isolation of spontaneous CsrRS mutants in the setting of invasive GAS infection, both in patients with severe invasive GAS infection and in experimental animals [18]–[21]. Exposure of wild type GAS to LL-37 or inactivation of CsrRS by mutation results in increased expression of factors that dramatically enhance GAS resistance to opsonophagocytic killing [12], [17]. These observations suggest that a physiologic role of CsrRS is to detect relatively low concentrations of LL-37 as a signal of mobilization of host defenses including the recruitment of phagocytic leukocytes and to trigger a global transcriptional response that enhances GAS resistance to phagocytosis.

We now report the results of further investigation that provides strong support for this hypothesis. LL-37 not only activates expression of the four previously identified loci, but also stimulates either activation or repression of multiple CsrRS-regulated genes. Signaling by LL-37 is dependent on CsrS, which is shown to have a surface-exposed domain on the bacterial cell. Transduction of the LL-37 signal requires specific domains of both CsrS and its cognate regulator CsrR to induce changes in gene expression. A critical consequence of LL-37-mediated CsrRS-signaling is enhanced resistance to phagocytic killing by human blood leukocytes, a bacterial phenotype that is central to both persistence of GAS in the human host and pathogenesis of invasive infection.

## Results

### LL-37 and Mg^2+^ have opposite effects on expression of multiple CsrRS-regulated genes

Earlier work by Gryllos *et al*. found that exposure of GAS to subinhibitory concentrations of LL-37 up-regulated expression of *hasB*, *spyCEP*/*scpC*/*prtS*, *mac*/*IdeS*, and *SPy0170*, genes that were shown previously to be down-regulated by extracellular Mg^2+^ in a CsrRS-dependent manner [11], [17]. Furthermore, the stimulatory effect of LL-37 on CsrRS-regulated gene expression could be blocked by high concentrations of Mg^2+^. These findings suggested the hypothesis that Mg^2+^ and LL-37 act as opposing extracellular signals for the CsrS sensor histidine kinase. To test if other CsrRS-regulated genes also respond to both stimuli, we investigated ten additional genes for their responsiveness to LL-37 and Mg^2+^ in GAS strain 854. This strain was chosen for further analyses because initial experimentation showed marked up-regulation of the four previously characterized CsrRS target genes by LL-37, signaling that was completely abrogated in an isogenic *csrS* deficient mutant [17]. Furthermore, strain 854 is representative of the widely disseminated M1T1 clone associated with invasive GAS infections over the past three decades [22]–[25].

**Figure 1:**
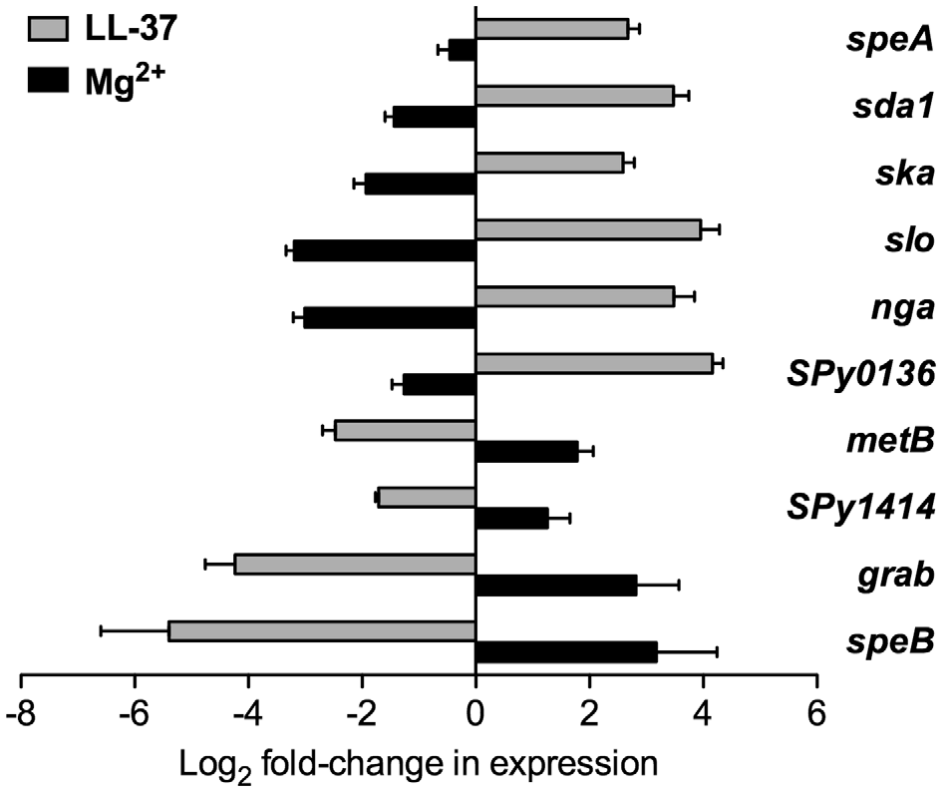
LL-37 and Mg^2+^ have opposite effects on expression of CsrRS-regulated genes. Expression of ten CsrRS*-*regulated genes in response to LL-37 or Mg^2+^ was quantified by qRT-PCR. Data represent mean fold-change ± SEM of gene expression in cultures grown in the presence of 100 nM LL-37 (grey bars) or 15 mM Mg^2+^ (black bars) relative to that in control cultures grown in unsupplemented medium (n = 3 – 5). Significant differences in response to LL-37 were found for all tested genes (P<0.008 for *speA*, *sda1*, *ska*, *SPy0136*, *metB*, *SPy1414*, *grab*; P<0.05 for *slo*, *nga* and *speB*). Significant differences in response to Mg^2+^ were found for all tested genes (P<0.02) except for *speA* and *SPy1414*.

In the present study, we found that exposure of strain 854 to 100 nM LL-37 resulted in up-regulation of *speA* (pyrogenic exotoxin A), *sda1* (DNase), *ska* (streptokinase), *slo* (streptolysin O), *nga* (NAD-glycohydrolase), and *SPy0136* (hypothetical protein; N.B., throughout this paper, unnamed open reading frames are designated by SPy numbers according to the SF370 or MGAS315 genome sequences [26], [27]), as assessed by quantitative RT-PCR (qRT-PCR) analysis of RNA samples from LL-37-treated and untreated bacteria (Figure 1). Culture of strain 854 in 15 mM Mg^2+^ had the opposite effect from that evoked by LL-37. That is, Mg^2+^ exposure resulted in down-regulation of these genes relative to their expression at baseline in unsupplemented medium (Figure 1). Conversely, expression of several genes in strain 854 was repressed by LL-37 and up-regulated by Mg^2+^. Genes in the latter category included *metB* (putative cystathionine beta-lyase), *SPy1414* (putative cation (potassium) transport protein), *grab* (protein G-related α_2_-macroglobulin-binding protein), and *speB* (cysteine protease) (Figure 1).

To verify that the changes in gene expression observed in response to LL-37 resulted in corresponding changes in production of the encoded proteins, we assayed four representative virulence determinants from this group of CsrRS-regulated genes. Growth of strain 854 in the presence of LL-37 resulted in marked increases in SLO and NADase and repression of SpeB, as assessed by western blot, and increased DNase activity (Figure S1). DNase activity associated with invasive M1T1 isolates such as strain 854 has been shown to be due predominantly to the enzyme encoded by the prophage-associated *sda1* gene (also called *sdaD2*), a member of the CsrRS regulon [28]. These results corroborate the qRT-PCR data and, together, they extend earlier findings that LL-37 can up-regulate gene expression to include several additional CsrRS-controlled genes. Moreover, they show that expression of certain CsrRS-regulated genes is repressed, rather than stimulated, by LL-37. For both categories of genes, the effect of Mg^2+^ is opposite to that of LL-37, an observation that supports the hypothesis that the two molecules act as functionally antagonistic stimuli for signaling through CsrRS.

### CsrS is associated with the cell membrane and includes a surface-exposed domain

**Figure 2:**
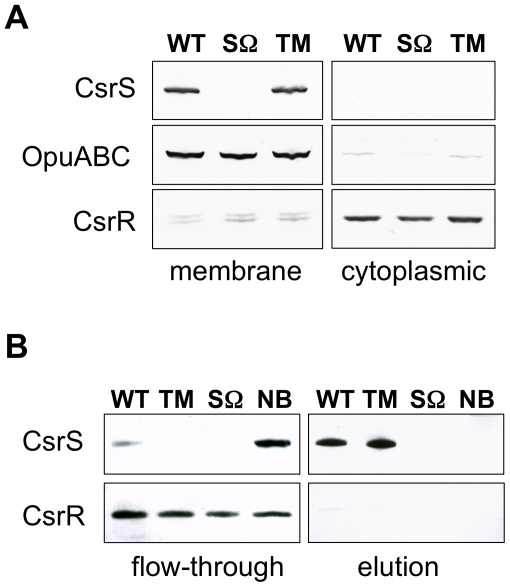
CsrS is associated with the cell membrane and contains a surface-exposed domain. A) Western blot analysis of membrane and cytoplasmic fractions isolated from whole cell lysates of GAS wild type strain 854 (WT), isogenic *csrS* deficient mutant strain 854*csrS*Ω (SΩ), and 854*csrS*_TM_ (TM) that expresses CsrS with 3 point mutations in the predicted extracellular domain. Specific antisera against CsrS, an unrelated membrane protein OpuABC, and CsrR were used to detect the respective proteins in both fractions. B) Biotin labeling via a disulfide linker of surface-exposed proteins in whole cells of wild type strain 854 (WT), 854*csrS*Ω (SΩ) and 854*csrS*_TM_ (TM). After lysis of labeled cells, biotinylated proteins were captured on a NeutrAvidin column and then eluted by reducing the disulfide linker. Specific antisera detected CsrS in the eluted fraction, as expected for a surface-exposed protein, and CsrR in the flow-through, as expected for a cytoplasmic protein. As a control, wild type 854 cells were treated similarly, but without biotin labeling (NB). Results shown in both panels are representative of at least two independent experiments.

The predicted histidine kinase CsrS is thought to represent a cell-surface sensor component of the CsrRS TCS that detects and responds to environmental signals. According to secondary structure and membrane protein model predictions, CsrS contains two membrane-spanning domains near the N-terminus that flank a predicted ECD of 151 amino acids [16]. To test these model predictions, membrane and cytoplasmic fractions of wild type GAS 854 and control *csrS*-deficient strain 854*csrS*Ω were isolated from whole cell lysates, fractionated by SDS-PAGE, and analyzed by western blot with anti-CsrS serum. CsrS was found exclusively in membranes of wild type bacteria and, as expected, was absent from *csrS* mutant preparations (Figure 2A). Like CsrS, the unrelated membrane protein OpuABC [11] was also in wild type 854 membranes, but not in the cytoplasmic fractions (Figure 2A). Consistent with its predicted cytosolic localization, the CsrR protein was mainly detected in the cytoplasmic fraction. These results localized CsrS to the GAS cell membrane.

In order to test whether CsrS is accessible to signaling molecules in the extracellular environment, we labeled proteins exposed on the bacterial surface with biotin via a disulfide linker. Biotinylated proteins were isolated from bacterial cell lysates using NeutrAvidin resin affinity chromatography. Resin-bound proteins were released by reduction of the disulfide bond linking biotin to the GAS surface proteins, fractionated by SDS-PAGE, and analyzed by western blot with CsrS antiserum. CsrS was detected predominantly in this eluted fraction (Figure 2B), a result that indicates CsrS was accessible to biotinylation, i.e., that a portion of the protein is exposed to the extracellular environment. CsrR, used here as a control cytosolic protein, did not react with biotin, and was detected only in the unbound flow-through fraction (Figure 2B). These data demonstrate that CsrS is a membrane-associated protein and includes a surface-exposed domain, conclusions consistent with our hypothesis that the ECD of CsrS functions as the sensor domain for environmental signals.

### A cluster of acidic amino acid residues in the CsrS ECD is critical to LL-37 signaling

**Figure 3:**
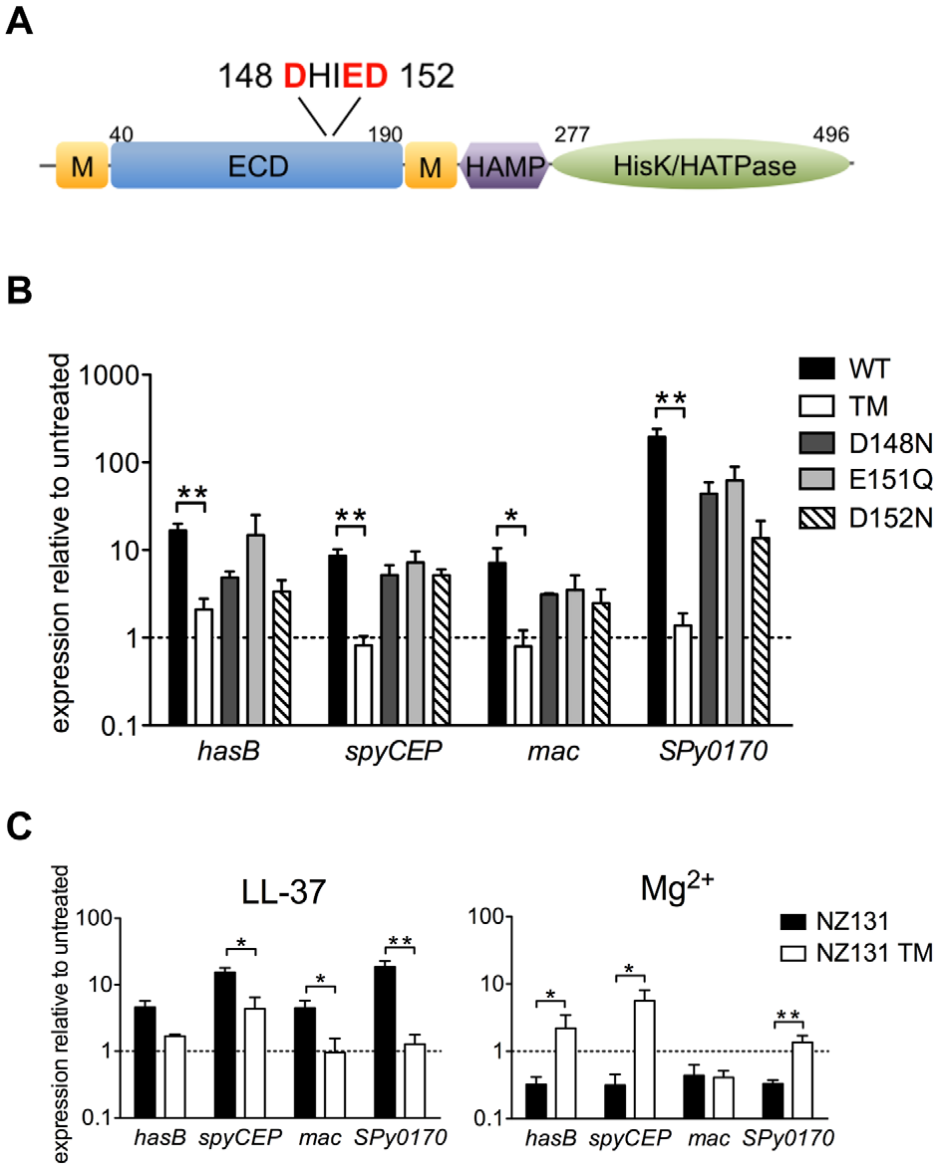
LL-37 and Mg^2+^ signaling of CsrRS-regulated genes involves a cluster of negatively charged amino acid residues located in the CsrS extracellular domain. A) Schematic representation of predicted CsrS protein domains. CsrS consists of two membrane-spanning domains (M), an extracellular domain (ECD), a cytosolic HAMP domain, a histidine kinase domain (HisK) and ATPase domain (HATPase). Acidic amino acids in the ECD replaced by uncharged residues in isogenic mutant strains are indicated in red. B) LL-37 stimulation of gene expression in wild type strain 854 (WT), isogenic *csrS* triple point mutant 854*csrS*_TM_ (TM), and isogenic *csrS* single point mutants 854*csrS*_D148N_ (D148N), 854*csrS*_E151Q_ (E151Q), and 854*csrS*_D152N_ (D152N). Expression of *hasB, spyCEP, mac,* and *SPy0170* was measured by qRT-PCR. C) LL-37 and Mg^2+^ responses in M-type 49 strain NZ131 and its isogenic *csrS* triple point mutant NZ131_TM_ (NZ131 TM). For panels B and C, data represent mean ratios ± SEM of gene expression in strains grown in the presence of 100 nM LL-37 (panel B; panel C, left) or 15 mM Mg^2+^ (panel C, right) compared to control cultures of the same strain grown in unsupplemented THY medium (n = 3 – 5). A broken line denotes a ratio of 1, which indicates no change in expression relative to that in unsupplemented medium. Asterisks denote significant differences between wild type and *csrS* triple point mutant (* P<0.05, ** P<0.006).

We noted previously that the predicted ECD of CsrS includes a cluster of negatively charged amino acids that corresponds to a similar cluster in PhoQ of *S. typhimurium* and *E. coli* implicated in binding of cationic ligands [17], [29], [30]. Preliminary experiments using wild type or mutant forms of *csrS* to complement *in trans* a *csrS* mutant of M-type 3 GAS strain DLS003 suggested that three charged residues in the ECD were required for LL-37 signaling. However, these experiments were not definitive as the level of CsrS protein expressed from the mutant construct was higher than that observed in the wild type strain [17]. To examine more thoroughly the role of the predicted CsrS ECD in LL-37 sensing by CsrS, we introduced point mutations into the chromosomal *csrS* locus of GAS strain 854 by allelic replacement. Four independent mutant strains were constructed in which one or all three negatively charged amino acids localized in a small cluster of acidic residues (148**D**HI**ED**152, Figure 3A) were substituted with similar uncharged residues (D148N, E151Q or D152N). Mutation of these three amino acids did not affect expression levels or surface localization of mutant CsrS, as similar quantities of CsrS were detected in western blots of membrane fractions obtained from the *csrS* triple point mutant strain 854*csrS*_TM_ and wild type 854 (Figure 2A), and similar amounts of CsrS were labeled by biotinylation on the mutant strain cell surface (Figure 2B). The four resulting isogenic *csrS* mutants 854*csrS*_D148N_, 854*csrS*_E151Q_, 854*csrS*_D152N_, and 854*csrS*_TM_ were tested for LL-37-mediated up-regulation of *hasB*, *spyCEP*, *mac,* and *SPy0170* expression. Wild type strain 854 and each of the mutant strains were grown to early exponential phase in the presence or absence of 100 nM LL-37, and gene expression was assessed by qRT-PCR. In contrast to wild type, the isogenic *csrS* triple mutant showed little or no change in gene expression in response to LL-37 (Figure 3B). The *csrS* mutants with single amino acid substitutions (D148N, E151Q or D152N) all showed moderate LL-37-mediated up-regulation of the four target genes, but less than that observed in wild type (Figure 3B). Mutation of this region of the CsrS ECD also abrogated or severely blunted the effect of Mg^2+^ to repress, or in the case of *grab*, to activate, CsrRS-regulated gene expression (Figure S2).

The results above provide evidence that the mutated cluster of acidic residues in the predicted ECD is critical for LL-37 and Mg^2+^ signaling through CsrS in strain 854. To confirm these findings and to test their generality for other GAS strains, we constructed an analogous *csrS* triple point mutant of M-type 49 strain NZ131 and examined its response to LL-37 by qRT-PCR. Similar to the results in the 854 background, we observed almost complete loss of LL-37-stimulated up-regulation of *hasB*, *mac,* and *SPy0170* in NZ131*csrS*_TM_, and a marked reduction in *spyCEP* up-regulation (Figure 3C, left panel). In wild type NZ131, expression of these four genes was repressed during growth in 15 mM Mg^2+^, but no such repression was observed for *hasB*, *spyCEP,* or *Spy0170* in the NZ131*csrS*_TM_ (Figure 3C, right panel). Thus, similar findings in two independent strain backgrounds highlight the importance of a small cluster of negatively charged amino acids in the predicted CsrS ECD in LL-37 and Mg^2+^ signaling through CsrRS.

### The pattern of gene regulation in 854*csrS*_TM_ is consistent with constitutive activation of CsrS autokinase activity

During characterization of the *csrS* triple mutant, we noted that mutant bacteria formed compact, glossy colonies similar to wild type 854, but distinctly different from the mucoid colony appearance of 854*csrS*Ω lacking CsrS. To verify that the distinctive colony morphology reflected a difference in capsule gene expression, we compared relative expression of *hasB* (from the hyaluronic acid capsule biosynthetic operon) in the three strains. As expected, in the absence of supplemental Mg^2+^ or LL-37, expression of *hasB* was increased more than 50-fold in strain 854*csrS*Ω relative to that in wild type 854, whereas *hasB* expression in the *csrS* triple point mutant was actually reduced by 40% compared to wild type (Table 1). This finding of reduced capsule gene expression suggested that the ECD mutations in the triple mutant resulted not only in refractoriness to regulation by LL-37, but also in increased activity of the CsrR response regulator, presumably by enhancing its phosphorylation in the absence of signaling from an external ligand. Such an effect could result from increased autokinase activity of CsrS or reduced phosphatase activity of CsrS for phospho-CsrR. To test this hypothesis, we compared expression of additional CsrRS-regulated genes in the *csrS* triple mutant relative to wild type 854. As observed for *hasB*, in the absence of supplemental Mg^2+^ or LL-37, expression of *spyCEP*, *mac,* and *SPy0170* was down-regulated in the triple mutant compared to wild type, whereas expression of each of these genes was significantly up-regulated in 854*csrS*Ω relative to wild type expression levels (Table 1). Furthermore, expression of *grab* was increased in the triple mutant relative to wild type levels (data not shown). As *grab* is activated by supplemental Mg^2+^ and repressed by LL-37 (Figure 1), this result is also consistent with the proposed model of increased CsrR activity in the triple mutant.

**Table 1:** Effect of *csrS* and *csrR* mutations on expression of CsrRS-regulated genes in GAS strain 854 under standard growth conditions.

	TM^a^	*csrS*Ωa,b	H280Aa,b	H280A TMa,b	Δ*csrR* a,b
*hasB*	0.6±0.1	53±11	42±7	34±2	66±10
*spyCEP*	0.5±0.0	209±5	210±5	219±11	126±12
*mac*	0.8±0.2	38±5	54±3	59±25	196±32
*SPy0170*	0.6±.1	496±82	914±327	632±209	6497±1235

Strain designations: TM, 854*csrS*_TM_; *csrS*Ω, 854*csrS*Ω; H280A, 854*csrS*_H280A_; H280A TM, 854*csrS*_H280A,TM_; *csrR*, 854Δ*csrR*.

a Transcript abundance for 854 *csrS* and *csrR* mutant strains grown to early exponential phase in unsupplemented THY medium relative to that in wild type strain 854. Expression of *hasB*, *spyCEP*, *mac*, and *SPy0170* was quantified by qRT-PCR. Values represent mean ratios ± SEM of three independent experiments performed in triplicate.

b Expression level of the four genes is significantly different (P<0.001) from that in wild type strain 854.

To test directly whether the altered ECD of the triple mutant changed gene regulation by affecting autokinase activity of CsrS, we inactivated the kinase by replacing the active site histidine residue with alanine (H280A). As expected, when introduced in strain 854, this mutation resulted in a mucoid colony morphology, and the mutant strain 854*csrS*_H280A_ displayed marked up-regulation of CsrRS-repressed genes in a pattern very similar to that observed in 854*csrS*Ω (Table 1). Similarly, introduction of the H280A mutation into the CsrS triple mutant resulted in mucoid colonies and a comparable derepression of CsrRS-repressed genes as in 854*csrS*Ω and in 854*csrS*_H280A_ (Table 1). Since mutation of the active site histidine of CsrS abrogated the suppressive effect of the ECD triple mutant, the most parsimonious model is that these mutations in the ECD affect gene expression by altering the autokinase activity of CsrS. While an effect on phosphatase activity is not excluded by these experiments, the results suggest strongly that the ECD triple point mutant expresses a constitutively active CsrS histidine kinase that is relatively refractory to signaling induced by external stimuli.

### Signaling through CsrRS modulates GAS resistance to opsonophagocytic killing, a key feature of invasive disease isolates

CsrRS regulates the expression of several genes that encode products implicated in GAS resistance to opsonophagocytic killing and cytotoxicity: *hasABC*, *slo, nga, spyCEP, sda1*, *mac,* and *speB*. Upregulation of antiphagocytic factors by host LL-37 is expected to enhance virulence in vivo; however, testing this hypothesis directly in an animal model is not possible, currently, since cathelicidins of other mammalian species do not share the CsrRS-signaling activity of human LL-37 [17]. Because in vitro resistance to phagocytic killing by human blood leukocytes correlates with GAS virulence in vivo [31], we used an in vitro assay to assess the effect of LL-37 on phagocytic resistance as a proxy for effects on in vivo virulence. As would be predicted by the effects of LL-37 on regulation of antiphagocytic factors, exposure of four unrelated wild type GAS strains to LL-37 increased resistance of all four strains to phagocytic killing in vitro [17]. Inactivation of CsrR in the M-type 3 strain DLS003 also resulted in increased resistance to phagocytic killing by human peripheral blood leukocytes, consistent with the marked up-regulation of CsrRS-regulated antiphagocytic factors in the mutant strain [12].

**Figure 4:**
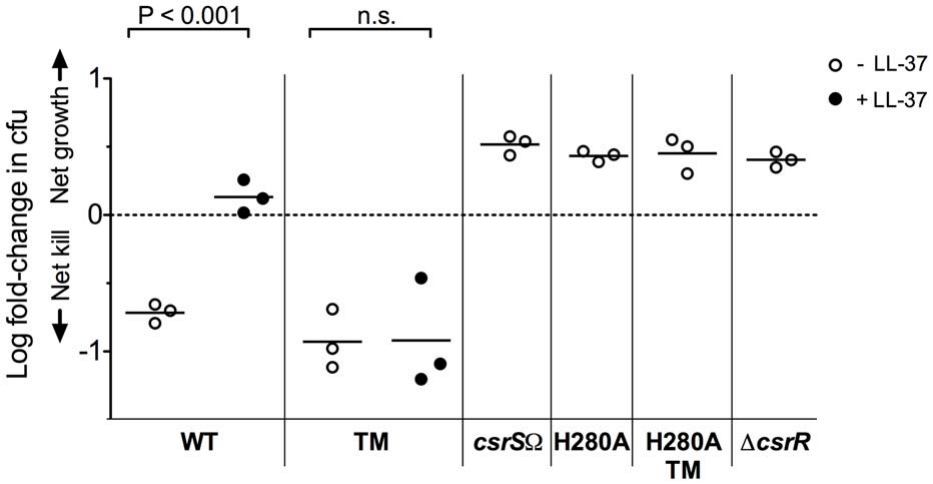
LL-37 signaling depends on a functional CsrS to induce GAS resistance to opsonophagocytic killing. Wild type strain 854 (WT), isogenic *csrS* mutants 854*csrS*_TM_ (TM), 854*csrS*Ω (*csrS*Ω), 854_H280A_ (H280A), and 854_H280A,TM_ (H280A TM), and isogenic *csrR* deletion mutant 854Δ*csrR* (Δ*csrR*) were grown in the absence (open symbols) or presence (filled symbols) of 100 nM LL-37. Bacteria were then mixed with human peripheral blood leukocytes for 1 h in the presence of 10% human serum as complement source. Values represent the log of mean fold-change in cfu. Each symbol represents a single experiment performed in duplicate. When exposed to LL-37, wild type 854 showed a significant increase in resistance to phagocytic killing compared to untreated bacteria (P<0.001), whereas the isogenic *csrS* triple mutant (TM) did not (n.s.  =  not significant). Mutant strains 854*csrS*Ω, 854_H280A_, 854_H280A,TM_, and 854Δ*csrR* were highly resistant to phagocytic killing in the absence of supplemental LL-37.

Because deletion of CsrS results in a similar, although less marked, up-regulation of CsrRS-controlled genes, we expected that deletion of CsrS or inactivation of its histidine kinase activity would also lead to increased resistance to phagocytic killing. In vitro opsonophagocytic assays of 854*csrS*Ω and 854*csrS*_H280A_ confirmed these predictions: both mutant strains were highly resistant to phagocytic killing by human blood leukocytes in vitro similar to a Δ*csrR* mutant (Figure 4). In marked contrast, the *csrS* triple mutant was as susceptible to killing as wild type 854 in the absence of LL-37, but did not show any increase in phagocytic resistance in response to LL-37 unlike wild type 854 (Figure 4). These observations further support the proposed model that the *csrS* triple mutant exhibits constitutive activation of CsrS autokinase activity and tonic phosphorylation of CsrR. An important consequence is down-regulation of CsrRS-controlled antiphagocytic factors and hyporesponsiveness to the stimulatory effect of LL-37.

### A conserved residue in the CsrR receiver domain is required for LL-37 and Mg^2+^ signaling

**Figure 5:**
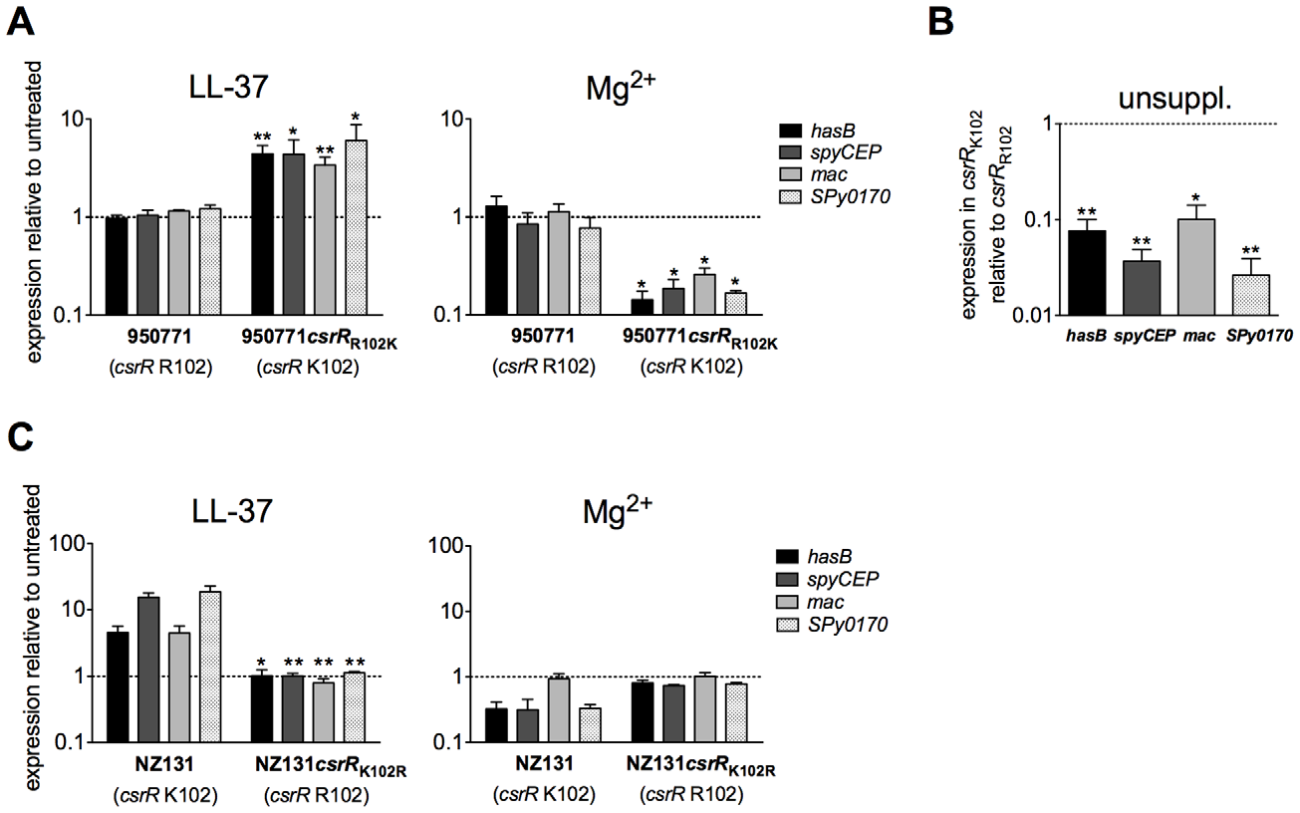
A lysine residue at position 102 is essential for CsrR to transduce LL-37 signaling. A) Regulation of CsrRS-dependent genes in 950771 and isogenic mutant strain 950771*csrR*_R102K_ in response to LL-37 and Mg^2+^. Gene expression was measured in strain 950771 with non-consensus CsrR (*csrR* R102) and in its isogenic mutant strain 950771*csrR*_R102K_ in which *csrR* residue 102 was changed from arginine to the consensus lysine (*csrR* K102); strains were grown in the presence of 100 nM LL-37 (left panel) or 15 mM Mg^2+^ (right panel). B) Comparison of baseline expression of *hasB, spyCEP, mac,* and *SPy0170* in isogenic mutant strain 950771*csrR*_R102K_ (consensus CsrR) with that in strain 950771 (non-consensus CsrR, *csrR* R102). Cultures were grown to early exponential phase in unsupplemented THY medium (unsuppl.). C) Mutation of *csrR* K102 in GAS wild type strain NZ131 prevents LL-37 and Mg^2+^ signaling. Gene expression was measured in NZ131 with consensus CsrR (*csrR* K102) and in its isogenic mutant strain NZ131*csrR*_K102R_ (*csrR* R102) grown in the presence of 100 nM LL-37 (left panel) or 15 mM Mg^2+^ (right panel). For all panels, data represent mean ratios ± SEM of at least three independent experiments done in triplicate. A broken line denotes a ratio of 1, which indicates no change in expression relative to control. Asterisks denote significant differences in expression between parental strain and respective mutant strains shown in each panel (* P<0.05, ** P<0.008).

The current working model for the CsrRS TCS is that of a classical sensor histidine kinase linked by phosphotransfer to a response regulator whose activity is controlled by its phosphorylation state. The experiments described above provide new evidence to support the surface location and stimulus-regulated histidine kinase activity of CsrS. We investigated also the role of CsrR in this model by further characterizing strain 950771, a GAS M-type 3 strain that exhibits a high level of capsular polysaccharide production that does not increase upon exposure to LL-37. Sequencing the *csrRS* locus in 950771 revealed a point mutation in *csrR* (K102R) in the highly conserved CsrR receiver domain [17]. The lysine residue that is mutated in 950771 is conserved not only in the *csrR* locus of all sequenced GAS strains, but also in response regulators of many TCS in a wide variety of bacterial species where it occupies a location near the conserved aspartic acid residue that is the site of phosphorylation [32]. In *E. coli* CheY, substitution of arginine for lysine at the corresponding site (K109R) did not prevent phosphorylation, but abrogated induction of tumbling motility that normally results from CheY phosphorylation, a finding interpreted to mean that the conserved lysine is required for phosphorylation to produce the active conformation of the response regulator [33]. To test whether K102 is required for GAS CsrR to transmit a signal from CsrS, we replaced R102 in strain 950771 with the consensus lysine residue (R102K). Whereas isolate 950771 (R102) showed no response to LL-37 or to Mg^2+^, strain 950771*csrR*_R102K_ restored both up-regulation of *hasB*, *spyCEP*, *mac,* and *SPy0170* in response to LL-37 and down-regulation in response to 15 mM Mg^2+^ (Figure 5A). In addition, correction of CsrR to the consensus K102 sequence markedly reduced expression of all four genes during growth in unsupplemented medium (Figure 5B), a result that implies that K102 is necessary for transduction of the signal mediated by tonic phosphorylation of CsrR by CsrS under standard laboratory growth conditions.

To further test whether the K102R CsrR mutation is sufficient to prevent CsrRS-mediated modulation of target gene expression, we also introduced the K102R mutation in wild type strain NZ131. In contrast to wild type NZ131 that exhibited a 2- to 25-fold increase in expression of *hasB*, *spyCEP*, *mac*, and *SPy0170* in response to 100 nM LL-37, mutant strain NZ131*csrR*_K102R_ showed no response (Figure 5C, left panel). Moreover, the mutant failed to repress expression of these genes in response to 15 mM Mg^2+^ (Figure 5C, right panel). Together, these results indicate that the conserved lysine residue at position 102 in CsrR is required for signal transduction from CsrS to modulate target gene expression in response to extracellular LL-37 or Mg^2+^.

## Discussion

Previous studies have demonstrated that CsrRS regulates expression of more than 100 GAS genes including those encoding many important virulence determinants [9]-[11]. In addition, evidence has been presented that elevated levels of extracellular Mg^2+^ result in down-regulated expression of CsrR-repressed genes, whereas exposure of GAS to subinhibitory concentrations of LL-37 has the opposite effect [11], [16], [17]. Results of the present study demonstrate the same reciprocal pattern of regulation by LL-37 and Mg^2+^ for an expanded repertoire of GAS genes. While the predominant pattern of regulation is one of up-regulation of gene expression by LL-37 and down-regulation by Mg^2+^, we report several instances of the opposite pattern, that is, repression of gene expression by LL-37 and activation by Mg^2+^. The current investigation provides new experimental evidence that supports a model of CsrRS as a classical TCS that responds to these environmental signals through modulation of CsrS autokinase activity, with downstream signaling that depends on phosphotransfer from CsrS to the CsrR transcriptional regulator. A critical consequence of CsrRS signaling by LL-37 is the coordinated modulation of expression of multiple genes in a fashion that dramatically increases GAS resistance to killing by phagocytes, a bacterial phenotype that enhances virulence and promotes invasive infection in vivo.

In addition to the previously demonstrated up-regulation by LL-37 of the hyaluronic acid capsule synthesis operon, *mac*/*IdeS* (Mac/IgG protease), and *spyCEP* (IL-8 protease), we found that LL-37 activated and Mg^2+^ repressed expression of genes encoding several important virulence factors including *ska* (streptokinase), *slo* (streptolysin O), and *nga* (NAD-glycohydrolase), as well as *speA* (pyrogenic exotoxin A) and *sda1* (DNase), two virulence determinants encoded by prophages associated with the invasive M1T1 GAS clonal group [34]. The opposite pattern of regulation was observed for *speB* (cysteine protease) and *grab* (protein G-related α_2_-macroglobulin-binding protein). Repressed expression of *speB* in response to LL-37 may also contribute to an invasive phenotype, as the *speB*-encoded cysteine protease has been proposed to degrade the anti-phagocytic M1 protein and to inactivate the *sda1* gene product, a DNase that itself enhances GAS virulence by degrading neutrophil extracellular traps (NETs) [21], [25], [35], [36].

Protein modeling of CsrS indicates the presence of two membrane-spanning regions that flank a domain predicted to form an extracellular loop that represents a potential site for interaction with environmental stimuli [12], [16]. In cell-fractionation experiments, we found that CsrS is physically associated with the bacterial cell membrane, as predicted by this model. Furthermore, CsrS on intact bacterial cells was accessible to biotin-labeling, a result that implies that a domain of the protein lies in the extracellular space. We also investigated the role in CsrS-mediated signal transduction of a small cluster of negatively charged amino acids in the CsrS ECD. Because a similar cluster of acidic residues has been implicated in binding of cationic ligands to *E. coli* and *S. typhimurium* PhoQ, we tested in GAS the effect of substituting uncharged amino acids for these three residues. While our intent had been to disrupt binding of Mg^2+^ and/or LL-37 to the CsrS ECD, we discovered that this relatively small alteration in the ECD not only abrogated ligand signaling, but also resulted in a global effect on the CsrRS regulon consistent with tonic activation of CsrS autokinase activity. Support for this hypothesis also came from the observation that the effects of the *csrS* ECD mutations were overridden by mutating the active site histidine of the CsrS kinase domain, a result that implies that the effects of the former mutations on target gene regulation are mediated through CsrS kinase activity. Thus, in the absence of increased extracellular Mg^2+^ or exposure to LL-37, expression of CsrR-repressed genes was reduced in the *csrS* triple mutant. The finding that neutralizing the charge of three amino acids in the CsrS ECD leads to an apparent activation of CsrS kinase activity and hyporesponsiveness to ligand signaling suggests that the mutations result in a conformational change in the cytoplasmic domain of CsrS that mimics that induced by binding of Mg^2+^ to the ECD. It is tempting to speculate that binding of Mg^2+^ and/or LL-37 to the same region of the ECD also modulates kinase activity by this mechanism, although attempts to demonstrate specific binding of either ligand to the ECD have, so far, been unsuccessful. The data summarized above suggest strongly that LL-37 signaling depends on direct interaction of the peptide and/or Mg^2+^ with the extracellular domain of CsrS. However, we cannot exclude an alternative signaling mechanism such as membrane disruption by LL-37 that secondarily results in altered CsrS autokinase activity.

We found that deletion of CsrS or inactivation of its kinase activity produced a similar pattern of altered gene expression as deletion of CsrR, although the magnitude of change in gene expression was somewhat smaller for some genes. These observations imply that, under laboratory growth conditions, CsrS activates CsrR, presumably by phosphorylation, increasing its activity as a transcriptional regulator. Activation of CsrR by CsrS can be increased by exposure to elevated extracellular Mg^2+^ or reduced by exposure to LL-37. The results discussed above support a model in which expression of the CsrRS regulon depends on the equilibrium between the phosphorylated and unphosphorylated states of CsrR. Increased extracellular Mg^2+^ or mutation of critical residues in the CsrS ECD increases CsrS phosphorylation and enhances phosphotransfer to CsrR, shifting the equilibrium toward phospho-CsrR with consequent repression of CsrR-repressed genes and activation of CsrR-activated genes. Conversely, exposure to LL-37 or deletion of CsrS shifts the equilibrium toward unphosphorylated CsrR, which is less active in regulating target promoters.

Transduction of these modulating signals to altered transcriptional regulation depends also on the presence of a conserved lysine residue at position 102 in the receiver domain of CsrR. A natural mutant with a conservative arginine substitution at this position was refractory to signaling by extracellular Mg^2+^ or exposure to LL-37 and exhibited a pattern of gene expression similar to that of a CsrR deletion mutant. These phenotypes were confirmed by repairing the natural mutant to the consensus K102 and by introducing the K102R mutation into an unrelated wild type strain. On the basis of these findings and work by others on the role of the corresponding lysine residue in bacterial TCS, we conclude that CsrR K102 is critical to transducing the signal of CsrR phosphorylation and to modulation of CsrR-mediated transcriptional regulation at target promoter sequences.

Several studies have documented the emergence of GAS strains with spontaneous inactivating mutations in CsrS or CsrR in the setting of invasive infection [18]–[20]. Because such mutants have a gene expression profile that results in a multifactorial enhancement of resistance to clearance by host phagocytes, these mutant variants have a strong selective advantage for survival in microenvironments such as the bloodstream or deep tissue sites where they are exposed to attack by host phagocytes. However, analysis of a collection of GAS pharyngeal isolates indicated that CsrRS mutants are distinctly rare in this setting, in marked contrast to isolates from patients with severe systemic infection [19]. The predominance of strains with a functional CsrRS system in the pharynx implies that CsrRS-mediated dynamic regulation of gene expression in response to environmental cues contributes to adaptation of GAS to its preferred environmental niche. During initial colonization, the low concentration of LL-37 on the resting pharyngeal epithelium is predicted to result in an intermediate level of CsrRS activation and a corresponding moderate expression of CsrRS-regulated virulence factors. This “colonizing” phenotype, however, can change quickly in response to increased local concentrations of LL-37. The striking up-regulation of an antiphagocytic phenotype upon exposure to LL-37 enables the organism to maintain the capacity to arm itself against host effectors and thus resist clearance. The coordinated program of altered gene expression induced by LL-37 signaling can tip the balance of pathogen-host interaction from one of asymptomatic colonization to uncontrolled invasive infection. Paradoxically, secretion of LL-37 from injured epithelial cells or from degranulation of recruited neutrophils as part of the host innate immune response may trigger local or systemic invasion by GAS as a result of CsrRS-mediated virulence factor expression.

## Methods

### Ethics statement

The human subjects aspects of this study were approved by the institutional review board of Children's Hospital Boston. Written informed consent was provided by study participants.

### Bacterial strains and growth conditions

Wild type GAS strains used in this study and isogenic mutants derived from them are described in Table 2. GAS M-type 1 strain 854 is a clinical isolate from a patient with a retroperitoneal abscess [17]. GAS M-type 49 strain NZ131 is a skin isolate from a patient with glomerulonephritis [37]. GAS strain 950771 is an M-type 3 clinical isolate from a child with necrotizing fasciitis and sepsis [38]. GAS strains were grown at 37°C in Todd-Hewitt broth (Difco) supplemented with 0.5% yeast extract (THY) or on THY agar or trypticase-soy agar (BD Bioscience) supplemented with 5% defibrinated sheep blood. *Escherichia coli* (*E. coli*) strains DH5α (New England Biolabs) and StrataClone (Stratagene) were used for cloning. Recombinant protein overexpression for antisera production was carried out using *E. coli* strain BL21(DE3) (Novagen). Antibiotics were used when necessary at the following concentrations: for GAS, erythromycin 1 µg/ml; for *E. coli*, erythromycin 200 µg/ml, kanamycin 50 µg/ml, carbenicillin 100 µg/ml, and ampicillin 100 µg/ml.

**Table 2:** GAS strains used in this study.

Strain	Source or reference	*csrS* or *csrR* genotype
854 (M-type 1)	[17]	wild type
854*csrS*Ω	[17]	*csrS*Ω
854*csrS*_D148N_	This study	*csrS*(D148N)
854*csrS*_E151Q_	This study	*csrS*(E151Q)
854*csrS*_D152N_	This study	*csrS*(D152N)
854*csrS*_TM_	This study	*csrS*(D148N,E151Q,D152N)
854*csrS*_H280A_	This study	*csrS*(H280A)
854*csrS*_H280A,TM_	This study	*csrS*(D148N,E151Q,D152N,H280A)
854Δ*csrR*	This study	Δ*csrR*
950771 (M-type 3)	[38]	*csrR*(R102)
950771*csrR*_R102K_	This study	*csrR*(R102K)
NZ131 (M-type 49)	[37]	wild type
NZ131*csrS*_TM_	This study	*csrS*(D148N,E151Q,D152N)
NZ131*csrR*_K102R_	This study	*csrR*(K102R)

### RNA isolation and qRT-PCR

GAS cultures were grown in THY broth supplemented with or without 100 nM LL-37 or 15 mM MgCl_2_, and cells were harvested either at early exponential (A_600 nm_ 0.25), mid-exponential (A_600 nm_ 0.5), late exponential (A_600 nm_ 0.8) or early stationary (A_600 nm_ ∼1) growth phase. Total RNA extraction from bacterial cells was performed as described [11] during the growth phase at which target gene expression was maximal. RNA concentration and purity were determined using a NanoDrop spectrophotometer ND-1000 (Thermo Fisher Scientific). Quantitative RT-PCR was performed on an ABI PRISM 7300 Real-Time PCR system (Applied Biosystems) using the QuantiTect SYBR Green RT-PCR kit (Qiagen). Primers used are listed in Table S1. Expression level of each target gene was normalized to *recA* (*spyM3_1800*/*SPy2116*) and analyzed using the ΔΔCt method as described [11]. Replicate experiments were performed from at least three independent RNA preparations in triplicate. Statistical analysis was performed using the paired Student's t-test for expression level comparison under different growth conditions in a single strain and the unpaired t-test for testing differences between strains.

### LL-37 synthesis

The human cathelicidin LL-37 (a gift of Robert I. Lehrer, UCLA, CA, USA) was synthesized as described previously and its purity was confirmed by high-performance liquid chromatography and mass spectrometry [39].

### Construction of recombinant plasmids for GAS mutagenesis

To introduce single point and triple point mutations in the CsrS ECD region, vector pORI*csrS* containing the wild type *csrS* sequence [11] served as template to amplify the entire plasmid by PCR with primer pair HTW 13/14 for *csrS*(D148N) substitution, HTW 15/16 for *csrS*(E151Q) substitution, HTW 17/18 for *csrS*(D152N) substitution, or csrS418-F(muNHIQN)/csrS480-R(muNHIQN) for *csrS* triple point substitution (D148N,E151Q,D152N) as described in the Quikchange site-directed mutagenesis protocol (Stratagene). From the resulting plasmids, *csrS* fragments used for allelic replacement were PCR-amplified with Phusion high-fidelity DNA polymerase (Finnzymes, New England Biolabs) by using primers csrS-F(PshAI) and rt0245-R and cloned into vector pSC-B (StrataClone blunt PCR cloning kit, Stratagene).

To introduce a *csrS* H280A mutation into GAS strain 854 and into the isogenic triple mutant strain 854*csrS*_TM_, a *csrS* fragment was amplified by PCR from wild type 854 chromosomal DNA with primer pair 5005_149F/5005_1204R and cloned into pGEM-T (Promega). The resulting plasmid was used for Quikchange site-directed mutagenesis to *csrS* H280A with primer pair H280A-F/H280A-R.

For generating a *csrR* deletion mutant in strain 854 (854Δ*csrR*), an overlap PCR using Phusion DNA polymerase was performed of the region upstream of *csrR* with primer pair Reg1P-F/HTW52 and the region downstream of *csrR* including about 500 bp of *csrS* with primer pair HTW 53/54. The hybridized strands of the two resulting PCR products were used as a template for the second PCR amplifying a 1 kb fragment encompassing a CsrR deletion of amino acids 3–223. The final product was ligated into vector pSC-B.

Subsequently, the *csrS* or Δ*csrR* fragments described above were released from pSC-B or pGEM-T by SalI/BamHI digestion and were subcloned into the temperature-sensitive shuttle vector pJRS233 [40].

To introduce the consensus CsrR (*csrR*(K102)) into GAS strain 950771 and non-consensus CsrR (*csrR*(R102)) into GAS strain NZ131, *csrR* R102 was amplified from 950771 chromosomal DNA was amplified by using Platinum Taq high fidelity DNA polymerase (Invitrogen) and primers CsrP-F and csrS176-R. The PCR product was cloned into pGEM-T and then subcloned into pJRS233 using PstI and XbaI restriction sites to generate pJRS-*csrR*(R102). Resulting plasmid pJRS-*csrR*(R102) was then used for Quikchange site-directed mutagenesis to convert *csrR*(R102) to *csrR*(K102) by using primer pair HTW 71/72, creating plasmid pJRS-*csrR*(K102). All primers are described in Table S1.

### Allelic exchange mutagenesis in GAS

Recombinant pJRS233 shuttle plasmids were electroporated into GAS strains 854, 854*csrS*_TM_, NZ131, or 950771 and then subjected to allelic gene replacement as described [38]. To confirm the genotype of mutant strains, *csrR* and *csrS* loci were PCR-amplified with Easy-A high fidelity DNA polymerase (Stratagene) from chromosomal DNA and the sequences confirmed by DNA sequencing (DNA Sequencing Core, Brigham and Women's Hospital, Boston, MA, USA).

### Preparation of GAS cell membrane and cytoplasmic fractions

Cultures of 854, 854*csrS*Ω, and 854*csrS*_TM_ were grown in THY at 37°C to an A_600 nm_ of ∼0.4, cells were collected (1250 × *g*, 8 min) and washed once with 10 mM Tris-HCl, pH 8.0, and resuspended in 360 µl hypotonic TEG buffer (10 mM Tris-HCl, 1 mM EDTA, 20% glucose, pH 8.0) supplemented with protease inhibitor cocktail III (Calbiochem). For peptidoglycan degradation, mutanolysin (∼500 units, Sigma) and lysozyme (∼17,700 units, Sigma) were added and samples were shaken at 1000 rpm at 37°C for 1 h in an Eppendorf thermomixer. Cells were washed once in 500 µl TEG buffer and resuspended in 500 µl TE buffer (10 mM Tris-HCl, 5 mM EDTA, pH 8.0) supplemented with protease inhibitor cocktail (Roche). Cells were lysed by ultrasonication (5×3 sec bursts on level 5, Sonic Dismembrator model 60, Fisher Scientific) on ice followed by centrifugation (Eppendorf 5417C 10,000 × *g*) for 20 min at 4°C to remove cell debris. Membranes were separated from the cytoplasmic fraction by ultracentrifugation of supernatants (Beckman Coulter Ultima, TLA-100.3 rotor) for 1 h at 90,000 × *g* at 4°C. Membranes and cytoplasmic fractions were resuspended in SDS-PAGE sample buffer and heated to boiling.

### Preparation of culture supernatant samples

GAS strain 854 was grown in liquid culture to A_600nm_ 0.7 (late exponential phase) or A_600nm_ 1.2 (stationary phase) in the absence or presence of 100 nM LL-37. Bacteria were removed by centrifugation (21,000 × *g*, 5 min). Cell-free supernatants were used for assays of DNase activity or mixed with sample buffer and heated to boiling before SDS-PAGE and western blot analysis.

### Western blotting

Samples were fractionated on 10% (membrane and cytoplasmic fractions) or 4–12% gradient (supernatant proteins) NuPAGE Novex Bis-Tris gels and then transferred to nitrocellulose membranes for western blotting as previously described [11]. Blots were incubated with specific rabbit antiserum against GAS CsrS ECD [11], CsrR, SLO [41], NADase [41], or SpeB (Toxin Technology, Sarasota, FL) at a 1∶1000 dilution, or with mouse antiserum against GAS membrane protein OpuABC (courtesy of Giuliano Bensi, Novartis Vaccines) at a 1∶3000 dilution, each followed by horseradish-peroxidase-linked secondary antibody [11]. Signal development was carried out using the SuperSignal West Pico chemiluminescence substrate (Thermo Scientific Pierce).

### Biotinylation of surface-exposed proteins in GAS

For surface biotinylation the Cell Surface Protein Isolation Kit (Pierce) was used according to the manufacturer's protocol with the following modifications. GAS were grown in 30 ml THY at 37°C to A_600 nm_ ∼ 0.3, cells were harvested (Centra CL3, Thermo IEC, 1250 × *g*, 8 min), washed twice in 1.5 ml PBS (Eppendorf 5417C, 9800 × *g*, 1 min), and resuspended in 1.5 ml Sulfo-NHS-SS-Biotin labeling solution. The biotinylation is reversible by cleavage of the disulfide bond in Sulfo-NHS-SS-Biotin. As a negative control, wild type 854 cells were incubated with PBS instead of biotin labeling solution. After 30 min agitation at 4°C, 100 µl quenching solution was added to treated cells and the cells were centrifuged at 6800 × *g* for 4 min. Cells were washed twice in 1.5 ml TBS (25 mM Tris-HCl, 0.15 M NaCl, pH 7.2) and frozen at −20°C. Frozen cells were resuspended in 250 µl lysis buffer supplemented with 2.5 µl protease inhibitor cocktail III (Calbiochem) and lysed by two rounds of ultrasonication (5×1 s bursts, level 1) with an incubation on ice in between. Lysates were centrifuged at 20,800 × *g* for 4 min at 4°C. Supernatants were incubated with 250 µl immobilized NeutrAvidin resin for 60 min in spin-columns with end-over-end rotation. Flow-through samples were retained and mixed with SDS-PAGE sample buffer. Resin was washed four times with 500 µl wash buffer supplemented with protease inhibitor and incubated with 200 µl SDS-PAGE sample buffer with 50 mM DTT for 60 min with end-over-end rotation at RT. Eluates were collected by brief centrifugation of uncapped spin-columns. Samples were heated at 95°C for 5 min and stored at −20°C until needed for western blot analysis. A 1∶3 mixture of antiserum to CsrS ECD and antiserum to N-terminal truncated CsrS was used to detect CsrS protein on the blots.

### Expression of recombinant Csr proteins components and development of antisera

Full-length CsrR and N-terminal truncated CsrS (CsrSΔ1-231) and were fused separately to a N-terminal His_6_ tag by cloning into overexpression vector pET-28a (Novagen) PCR-amplified DNA fragments obtained with primer pairs JL-48/JL-49 and HTW 37/46, respectively. Following overexpression by IPTG induction, recombinant proteins were affinity purified using Ni^2+^-NTA resin (Qiagen) under native conditions (His_6_-CsrR) or under denaturing conditions (His_6_-CsrSΔ1-231) according to the manufacturer's protocol. Purified proteins were used to immunize rabbits (LAMPIRE Biological Laboratories, Inc., Pipersville, Pennsylvania, USA). Reactivity of immune sera against CsrS or CsrR was evaluated by western blotting of GAS lysates.

### DNase activity assay

DNase activity in GAS culture supernatants was assayed as described by Aziz *et al.* with modifications [42]. Supernatants were diluted 1∶125 in sterile deionized water, and 10 µL samples were mixed with 1 µg plasmid DNA in 100 mM Tris, pH 7.5, supplemented with 1 mM CaCl_2_ and 1 mM MgCl_2_ in a 15 µL total reaction volume. Samples were incubated at 37°C for 20 min and then were stopped by the addition of 20 mM EDTA. Samples were analyzed on 1% agarose gels and DNA was visualized with SYBR Safe DNA stain (Invitrogen).

### Opsonophagocytosis assays

GAS resistance to phagocytic killing was evaluated by an in vitro assay as described [43]. In brief, GAS strains grown to early exponential phase with or without 100 nM LL-37 were mixed with freshly isolated human peripheral blood leukocytes at a multiplicity of infection of 3 – 4 in the presence of 10% human serum as complement source. Aliquots were withdrawn for quantitative culture immediately after mixing and after 1 h end-over-end rotation at 37°C. Results were reported on a log scale as the fold-change in cfu defined as the total cfu after incubation divided by the total starting cfu. Statistical significance of differences in the capacity of GAS strains to resist opsonophagocytic killing were evaluated by one-way ANOVA with Bonferroni's post-test analysis.

## Supporting Information

Figure S1 **Effects of LL-37 on gene transcription are reflected by expression levels of CsrRS-regulated gene products.** A) Western blots demonstrate increased amounts of SLO and NADase and reduced SpeB in culture supernatants of GAS strain 854 grown in the presence (+) of 100 mM LL-37 compared to supernatants from unsupplemented cultures (-). B) DNase activity reflecting production of Sda1, as assessed by degradation of plasmid DNA, was increased in culture supernatants of strain 854 grown in the presence (+) of LL-37 compared to supernatants from unsupplemented cultures (-). Lane C represents a control sample that contains an equivalent amount of plasmid DNA without culture supernatant added.
(TIF)

Figure S2 **Mg^2+^ signaling of CsrRS-regulated genes is impaired in strain 854** ***csrS*** **_TM_.** Data represent mean ratios ± SEM of gene expression as assessed by qRT-PCR in strain 854 (WT) or its isogenic *csrS* triple point mutant 854*csrS*TM (TM) grown in the presence of 15 mM Mg2+ compared to control cultures of the same strain grown in unsupplemented medium (n = 3 – 4). The broken line denotes a ratio of 1, which indicates no change in expression relative to that in unsupplemented medium. P<0.05 for comparison of Mg2+ response between 854 wild type and 854*csrS*TM for each of the four tested genes.
(TIF)

Table S1 Oligonucleotide primers used in this study.
(PDF)
