# Supplementary figures and images for: Signal Transduction through CsrRS Confers an Invasive Phenotype in Group A Streptococcus
Source: PLoS Pathog. 2011 Oct 27;7(10):e1002361. doi: 10.1371/journal.ppat.1002361 (PMC3203184; doi:10.1371/journal.ppat.1002361)

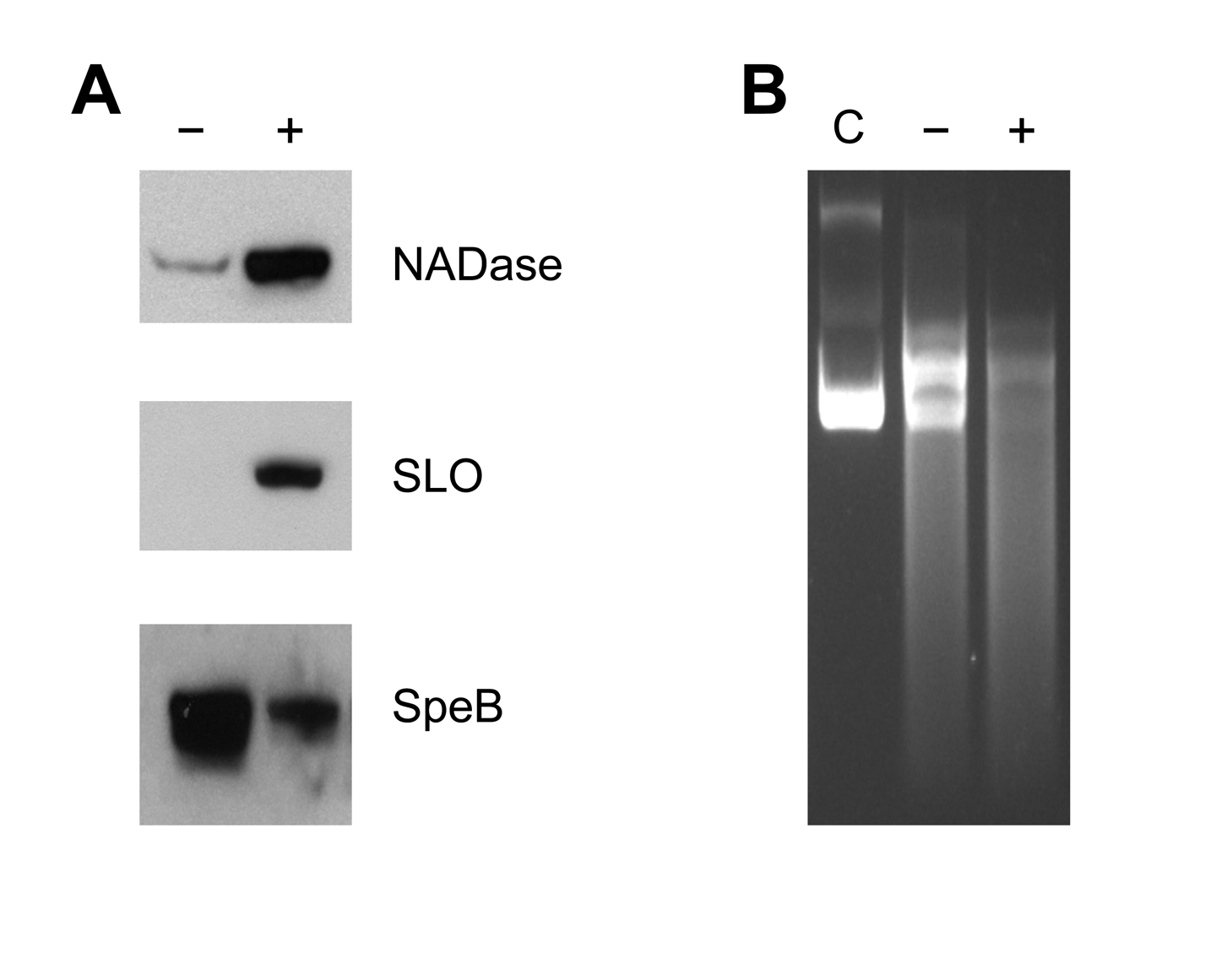

Supplement: Figure S1 — Effects of LL-37 on gene transcription are reflected by expression levels of CsrRS-regulated gene products. A) Western blots demonstrate increased amounts of SLO and NADase and reduced SpeB in culture supernatants of GAS strain 854 grown in the presence (+) of 100 mM LL-37 compared to supernatants from unsupplemented cultures (-). B) DNase activity reflecting production of Sda1, as assessed by degradation of plasmid DNA, was increased in culture supernatants of strain 854 grown in the presence (+) of LL-37 compared to supernatants from unsupplemented cultures (-). Lane C represents a control sample that contains an equivalent amount of plasmid DNA without culture supernatant added. (TIF) [file ppat.1002361.s001.tif]

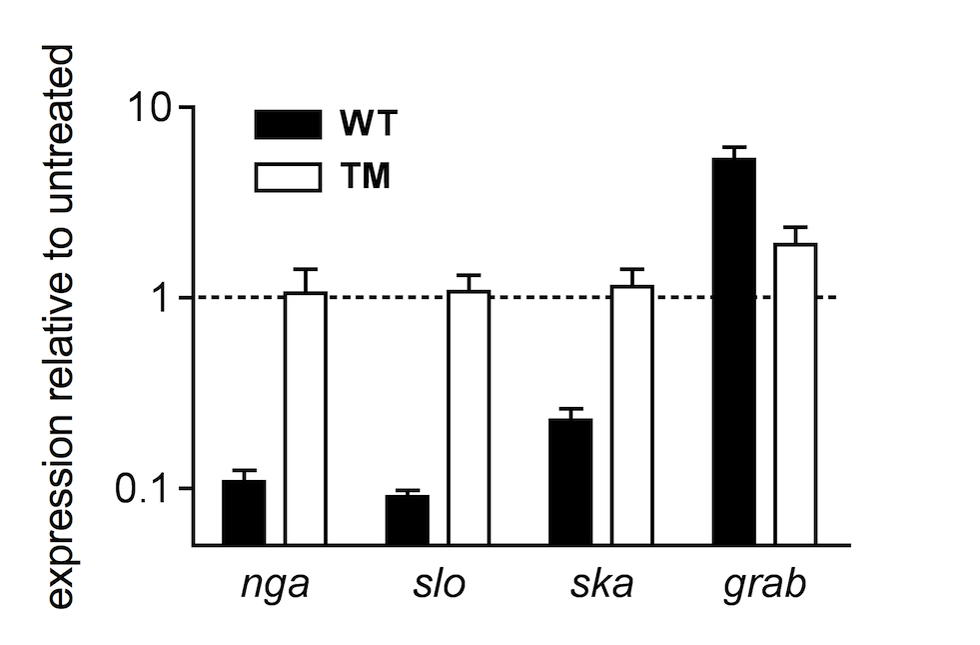

Supplement: Figure S2 — Mg2+ signaling of CsrRS-regulated genes is impaired in strain 854 csrS TM. Data represent mean ratios ± SEM of gene expression as assessed by qRT-PCR in strain 854 (WT) or its isogenic csrS triple point mutant 854csrSTM (TM) grown in the presence of 15 mM Mg2+ compared to control cultures of the same strain grown in unsupplemented medium (n = 3 – 4). The broken line denotes a ratio of 1, which indicates no change in expression relative to that in unsupplemented medium. P<0.05 for comparison of Mg2+ response between 854 wild type and 854csrSTM for each of the four tested genes. (TIF) [file ppat.1002361.s002.tif]
