# Supplementary material for: Signal Transduction through CsrRS Confers an Invasive Phenotype in Group A Streptococcus
Source: PLoS Pathog. 2011 Oct 27;7(10):e1002361. doi: 10.1371/journal.ppat.1002361 (PMC3203184; doi:10.1371/journal.ppat.1002361)
Supplement: Table S1 — Oligonucleotide primers used in this study. (PDF) [file ppat.1002361.s003.pdf]

Table S1. Oligonucleotide primers used in this study.

Primer[a]	DNA sequence 5'- 3'[b]	Position[c]	Reference
csrS418-F(muNHIQN)	ggtcctatcggtcgtgtgtatcac**a**a**t**catatc**c**a**ga**a tcagtatcgtgggttttccatgaca	428-480 of *csrS*	this study
csrS480-R(muNHIQN)	tgtcatggaaaacccacgatactg**a**t**t**ctggat**a**t**ga**t tgtgatacacacgaccgataggacc	480-428 of *csrS*	this study
HTW 12	gtcgtgtgtatcac**a**accatatcgaagatcag	428-459 of *csrS*	this study
HTW 13	ctgatcttcgatatggt**t**gtgatacacacgac	459-428 of *csrS*	this study
HTW 14	cacgaccatatc**c**aagatcagtatcgtgg	439-467 of *csrS*	this study
HTW 15	ccacgatactgatctt**g**gatatggtcgtg	467-439 of *csrS*	this study
HTW 16	cacgaccatatcgaa**a**atcagtatcgtggg	439-468 of *csrS*	this study
HTW 17	cccacgatactgat**t**ttcgatatggtcgtg	468-439 of *csrS*	this study
HTW 37	gcggcggtcgacct**cta**actctctttagactgg	*csrS*; SalI site, in bold stop codon of *csrS*	this study
HTW 46	gcgcgcatatgtctgaaaatcctaataatttaaacctg	starts *csrS* AA 232, NdeI site	this study
HTW 52	catatgacttatttctcacgaataacggtcatttatac caacccttatc	upstream *csrRS*	this study
HTW 53	cgttattcgtgagaaataagtcatatg	upstream *csrR*	this study
HTW 54	gaaagacttgaacatagcccac	downstream *csrS*	this study
HTW 71	cgactatattgtta**a**accgtttgccattg	*csrR*	this study
HTW 72	caatggcaaacggt**t**taacaatatagtcg	*csrR*	this study
JL-48	gtacgctagcatgacaaagaaaattttaattattgaag	*csrR*, NheI	this study
JL-49	caatctcgagacttatttctcacgaataacgtatcc	*csrR,* XhoI	this study
Reg1P-F	agagttggttgaagacaatctggtat	-365 to -341 of *csrR*	this study
CsrP-F (PstI)	aactgcagagctattctttgatatactctt	-224 to -203 nt from *csrR* start	this study
csrS176-R (XbaI)	gctctagatttacagcttgaaagactgactg	+154 to +176 nt *csrS*	this study
csrS-F (*Psh*AI)	acgacaagagtctcgaaaatcagaaacaaaaacagaag	*csrS*	this study
rt0245-R (*csrS*)	catgattgccatacggtcag	972-953	this study
5005_149F	ggatccgcagtcgagtcattaggagtgagc	286-310 of *csrS*	this study
5005_1204R	gtcgacccagcctgggtacttgttcg	1368-1351 of *csrS*	this study
H280A-F	gtgatgtcagcg**gc**tgaattacgaacaccg	825-855 of *csrS*	this study
H280A-R	cggtgttcgtaattca**gc**gctgacatcac	855-825 of *csrS*	this study

qRT-PCR primers			
rt0105-F (SPy0136)	acgacgggaaattttctcac	410-429	this study
rt0105-R (SPy0136)	aacttgggaatgatgctgct	595-576	this study
rt0132-F (SPy0170)	ttatgatcgcaactgctgctg	23-43	[11]
rt0132-R (SPy0170)	tcaggagcattttgtccgtag	128-108	[11]
rt0132-R4 (SPy0170)	ggaccagtgtgtccgtagaaa	137-117	[11]
rt0133-F (*metB*)	cagacttcgacgttccacaa	160-179	this study
rt0133-R (*metB*)	ataagccaccgcataagtcg	279-278	this study
rt0165-F1 (*nga*)[d]	acgttagccgcaaataccac	35-54	this study
rt0165-R1 (*nga*)[d]	tgtcgaccaatcgttgactt	186-167	this study
rt0167-F (*slo*)[d]	tacaaaacgacgttgggaca	1482-1501	this study
rt0167-R (*slo*)[d]	gatcacttttcgccaccatt	1626-1604	this study
rt0298-F (*prtS*)	cgcgttagccttaaaacagc	4447-4466	[11]
rt0298-R (*prtS*)	aggcaggctgacaacaacttc	4566-4546	[11]
rt0583-F (*mac*)	ccacagcagggaatatgcttca	290-311	[11]
rt0583-R (*mac*)	caaacatctgttcgccattg	406-387	[11]
rt0996-F (*speA2*)[d]	gggtgaccctgttactcacg	168-189	this study
rt0996-R (*speA2*)[d]	gcactcctttctgcattttca	380-361	this study
rt1032-F (*grab*)	ctgggaaaaagcagcaactc	786-805	[11]
rt1032-R (*grab*)	cgcagcagctgtaaagaatg	930-911	[11]
rt1078-F (SPy1414)	cgtgatgttggtgattttcg	1756-1775	this study
rt1078-R (SPy1414)	aaccaacgcattggactagc	1892-1873	this study
rt1415-F (*sda1*)[d]	agcatagccccaaaaatgtg	461-482	this study
rt1415-R (*sda1*)[d]	ctgcatcccacctttacgat	666-647	this study
rt1698-F (*ska*)	cgcaatgccacataaacttg	282-301	this study
rt1698-R (*ska*)	tcgcttgcaaaatcaatgac	395-376	this study
rt1742-F (*speB*)	accaaatcaaccgtagcgac	908-927	this study
rt1742-R (*speB*)	aggcatgtccgcctacttta	1024-1005	this study
rt1800-F (*recA*)	tgattctggtgcggttgatc	282-301	[11]
rt1800-R (*recA*)	atttacgcatggcctgactc	415-396	[11]
rt1852-F (*hasB*)	tccccaaacgctaattgaag	825-844	[11]
rt1852-R (*hasB*)	ttaaacggtaaaccccgact	952-933	[11]

[a] Unless designated otherwise, numbers preceded by "rt" or "SPy" refer to ORF numbers for M-type 1 SF370 [26] or M type 3 strain MGAS315 [27]; F = Forward; R = Reverse
[b] restriction endonuclease sites are underlined; nucleotide substitutions are shown in bold
[c] nucleotide position relative to the start codon
[d] numbers indicate M-type 1 MGAS5005 ORFs [25]
